# Retrograde–antegrade rendezvous recanalization for complete cervical esophageal obstruction after chemoradiotherapy: a novel organ-preserving approach

**DOI:** 10.1055/a-2837-9059

**Published:** 2026-05-13

**Authors:** Mitsuhiro Kono, Masaki Ominami, Daiki Kitagawa, Shunsuke Takahashi, Miyu Emoto, Shusei Fukunaga, Yasuhiro Fujiwara

**Affiliations:** 1 Department of Gastroenterology 12935 Osaka Metropolitan University Graduate School of MedicineOsakaJapan

**Fig. 1:**
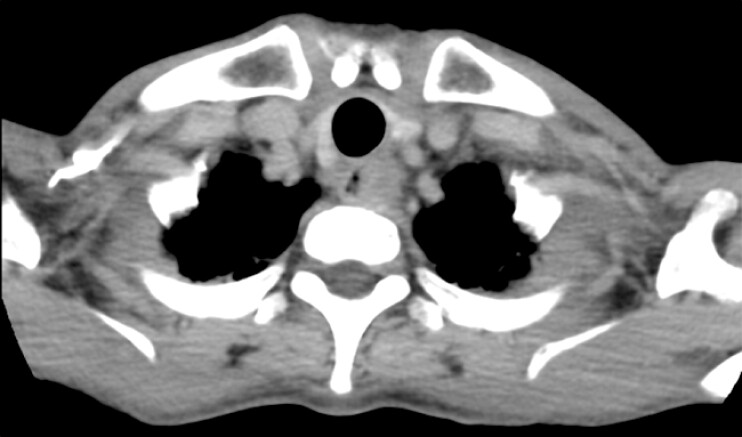
A CT scan revealed an image showing the cervical esophagus occupied by esophageal cancer. CT, computed tomography.

A 60-year-old woman had cT3N1M0 clinical Stage IIIb cervical esophageal cancer ( Fig. 1 ), desired larynx preservation, and opted for chemoradiotherapy (CRT). Before CRT, the patient presented with dysphagia, and because of an esophageal cancer-induced stricture, even a thin scope could not be passed. Therefore, gastrostomy was performed under CT guidance , followed by CRT with cisplatin and fluorouracil at a radiation dose of 60 Gy (30 fractions). While receiving nutritional support via a gastrostomy tube, the patient maintained minimal oral intake. Although complete remission was achieved with CRT, the patient could not receive oral intake.

**Fig. 2:**
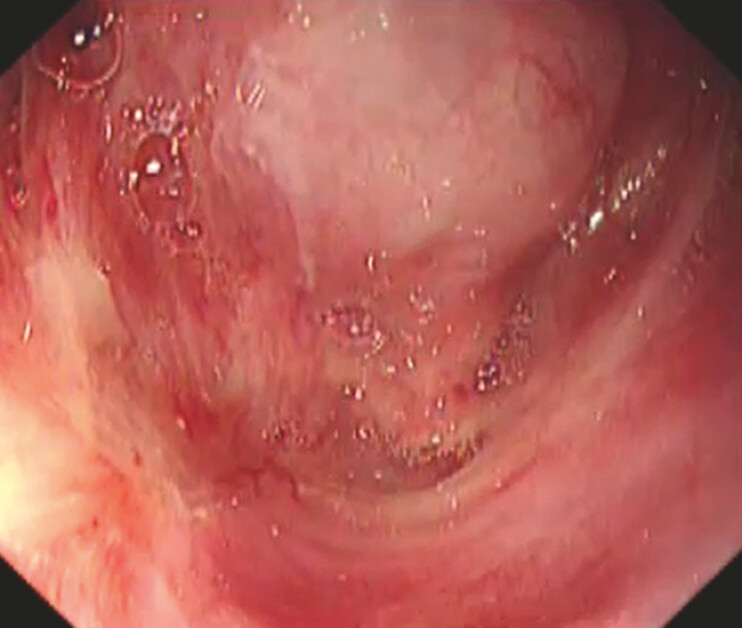
The esophageal entrance is completely obstructed by CRT-induced scarring. CRT, chemoradiotherapy.

An attempt was made to insert an endoscope for endoscopic balloon dilation (EBD), but the esophageal entrance was completely obstructed by CRT-induced scarring ( Fig. 2 ).

**Fig. 3:**
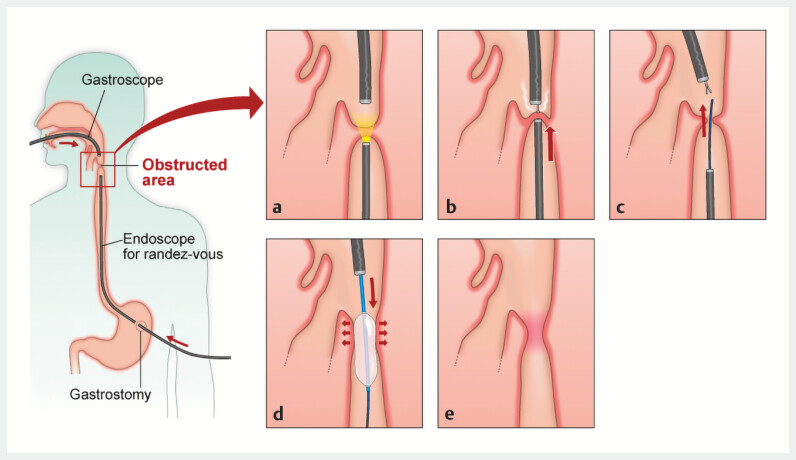
**a** The thin endoscope inserted through the gastrostomy illuminated the obstructed area using transmitted light. **b** Using forceps from the gastrostomy side, the obstruction point was compressed, and mucosal incision was performed using its protrusion as a landmark. **c** and **d** A guidewire was passed from the gastrostomy side toward the oral side, and a balloon was inserted using the rendezvous technique. **e** An image of the cervical esophagus after EBD. EBD, endoscopic balloon dilation. Source: Medical Fig.

**Fig. 4:**
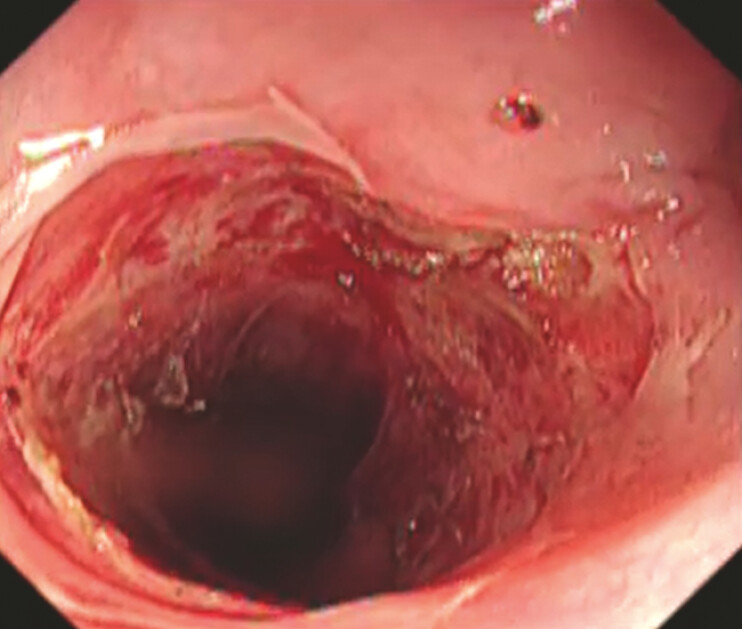
An image of the cervical esophagus after EBD. EBD, endoscopic balloon dilation.

**Fig. 5:**
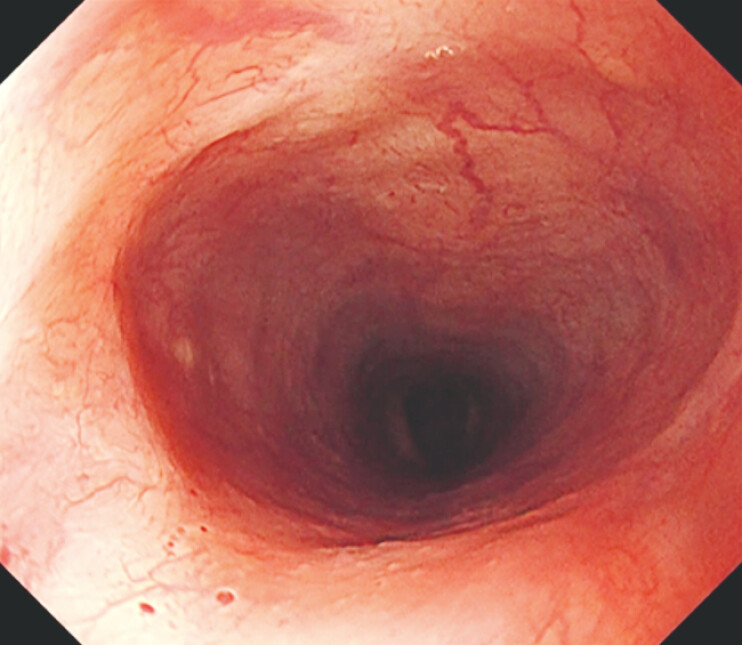
An image of the cervical esophagus 2 months after treatment. Oral steroids were administered, and the lumen remained open to approximately 12 mm.

Under general anesthesia, a thin endoscope was inserted retrogradely from the gastrostomy site to the cervical esophagus 1 . Relying on the forceps pressure applied from the anal side of the obstruction, a 5 mm incision was made from the oral side using a technique similar to radial incision and cutting 2 . After advancing the incision and confirming communication with the anal side, a 0.025-inch guidewire was pulled from the gastrostomy side to the pharyngeal side ( Video 1 ), and EBD was performed using the through-the-wire technique ( Video 1 and Fig. 3 ). After EBD using a 10–12 mm balloon, a lumen approximately 12 mm in diameter was achieved ( Fig. 4 ). Subsequently, steroid tapering therapy was used, and the lumen remained patent without re-obstruction ( Fig. 5 ). The patient could eat orally while undergoing periodic EBD.

In the through-the-wire technique, a guidewire is pulled from the gastrostomy side to the pharyngeal side.Video 1

EBD is performed to treat strictures after CRT for advanced esophageal cancer 3 4 . Complete cervical esophagus obstruction after CRT rarely occurs, necessitating pharyngolaryngoesophagectomy 5 . As in this case, performing the rendezvous technique from both the gastrostomy and oral sides prevents a highly invasive treatment.

Endoscopy_UCTN_Code_TTT_1AO_2AH
